# Predicting peripartum blood transfusion in women undergoing cesarean delivery: A risk prediction model

**DOI:** 10.1371/journal.pone.0208417

**Published:** 2018-12-14

**Authors:** Homa K. Ahmadzia, Jaclyn M. Phillips, Andra H. James, Madeline M. Rice, Richard L. Amdur

**Affiliations:** 1 Department of Obstetrics & Gynecology, Division of Maternal-Fetal Medicine, George Washington University, Washington, DC, United States of America; 2 Department of Obstetrics & Gynecology, Division of Maternal-Fetal Medicine and Department of Medicine, Division of Hematology, Duke University, Durham, NC, United States of America; 3 Department of Epidemiology and Biostatistics and the Biostatistics Center, George Washington University, Washington, DC, United States of America; 4 Department of Surgery, George Washington University, Washington, DC, United States of America; Federal University of Sergipe, BRAZIL

## Abstract

**Objective:**

There has been an appreciable rise in postpartum hemorrhage requiring blood transfusions in the United States. Our objective is to better define patients at greatest risk for peripartum transfusion at the time of cesarean in order to identify cases for early intervention and monitoring.

**Methods:**

Our study is a secondary analysis of a retrospective cohort study. Cases of intraoperative and immediate postpartum blood transfusion among women undergoing cesarean delivery were identified. Multivariable logistic regression models were used to identify antepartum and intrapartum risk factors that were independently associated with blood transfusion. A risk calculator was then developed to predict the need for transfusion.

**Results:**

Of 56,967 women, 1488 (2.6%) required any blood transfusion. The strongest risk factors for peripartum blood transfusion included anemia (odds ratio [OR] 3.7, 95% CI 3.3–4.3), abruption on presentation (OR 3.3, CI 2.6–4.1), general anesthesia (OR 5.2, CI 4.4–6.1) and abnormal placentation (OR 92.0, CI 57.4–147.6). An antepartum (model 1) and combined antepartum plus intrapartum risk model (model 2) were developed (model 1 AUC = 0.77, model 2 AUC = 0.83) and internally validated.

**Conclusions:**

Among women who required cesarean delivery, we were able to identify risk factors which predispose women to peripartum blood transfusion and developed a prediction model with good discrimination.

## Introduction

Massive obstetric hemorrhage contributes to over one half of the observed morbidity and mortality worldwide and is not limited to resource-poor settings [1–5]. Classically, severe postpartum hemorrhage (PPH) is defined as blood loss greater than 500cc in a vaginal delivery and greater than 1000cc in a cesarean delivery[6,7]. More recently, an estimated blood loss greater than 1000cc in either vaginal or cesarean delivery has been adopted by US national organizations as the definition of PPH[8]. Due to inconsistency in how postpartum hemorrhage is defined and lack of precision in the estimated blood loss, a more objective way to define morbidity associated with obstetric hemorrhage is the requirement of blood transfusion.

The rate of blood transfusion is estimated at 0.4–1.6% of all deliveries[8–11], however, data have emerged highlighting temporal trends toward increasing rates of PPH requiring transfusion [3,6,12–14]. Many risk factors for severe PPH have been established including race/ethnicity, multifetal gestation, birthweight, hypertensive disorders, abruption, labor induction, placental abnormalities, severe antenatal anemia, advanced maternal age, and general anesthesia[8,9,14–16]. Specifically, cesarean delivery (CD) is a predominant and independent risk factor for severe obstetric hemorrhage that has been well cited throughout the literature, and thus is the focus of this study.[9,14,15,17,18].

Delayed diagnosis and management of obstetric hemorrhage contributes greatly to maternal morbidity and mortality[5,7]. The World Health Organization advocates that with increased prevention measures and aggressive treatment, many deaths related to PPH could be avoided[5]. An important contribution to patient safety is identification of those at risk for obstetric hemorrhage and employment of preoperative preparedness protocols. In addition to standard protocols, patients who are at particularly high risk of hemorrhage may benefit from prevention strategies. For example, evidence suggests that administration of tranexamic acid prior to cesarean can reduce the risk of transfusion (1.9% vs. 5.7%, RR 0.33 95% CI 0.19–0.58)[19]. However, TXA is not yet routinely used in CD for postpartum hemorrhage prevention, but could be an adjunct medication for those we identify as high risk. Our study objective is to use population level data to develop and validate a prediction model for peripartum hemorrhage requiring blood transfusion to aid in prevention of postpartum transfusion in those undergoing CD.

## Materials and methods

This study is a secondary analysis of the Cesarean Registry, a *Eunice Kennedy Shriver* National Institute of Child Health and Human Development (NICHD) Maternal-Fetal Medicine Units (MFMU) Network registry. The Cesarean Registry is a multicenter observational study across 19 institutions initially designed to collect data on complications surrounding CD and trial of labor after prior cesarean (TOLAC)[20].

Data were collected from 1999 through 2002. From 1999 to 2000, patients were enrolled who underwent primary or repeat CD or vaginal birth after cesarean (VBAC). From 2001 to 2002, patients were only enrolled who underwent repeat CD or VBAC. Patients who met criteria for inclusion were all greater than 20 weeks gestation or delivered a fetus weighing ≥ 500 grams. Demographic, history, and delivery information were collected through chart review at the time of delivery by certified research personnel. Information on delivery course, neonatal outcomes, and complications were obtained through a complete chart review and discharge information. Each chart was reviewed 6 weeks after discharge to ensure all re-admissions and complications were identified. Through the Office of Human Research, The George Washington University Institutional Review Boards (IRBs) granted exempt status for this secondary analysis (IRB 061644) on June 29, 2016. All data were fully anonymized prior to accessing the information for the study and the committee waved requirement of informed consent for this retrospective analysis.

### Study population

**Fig 1:**
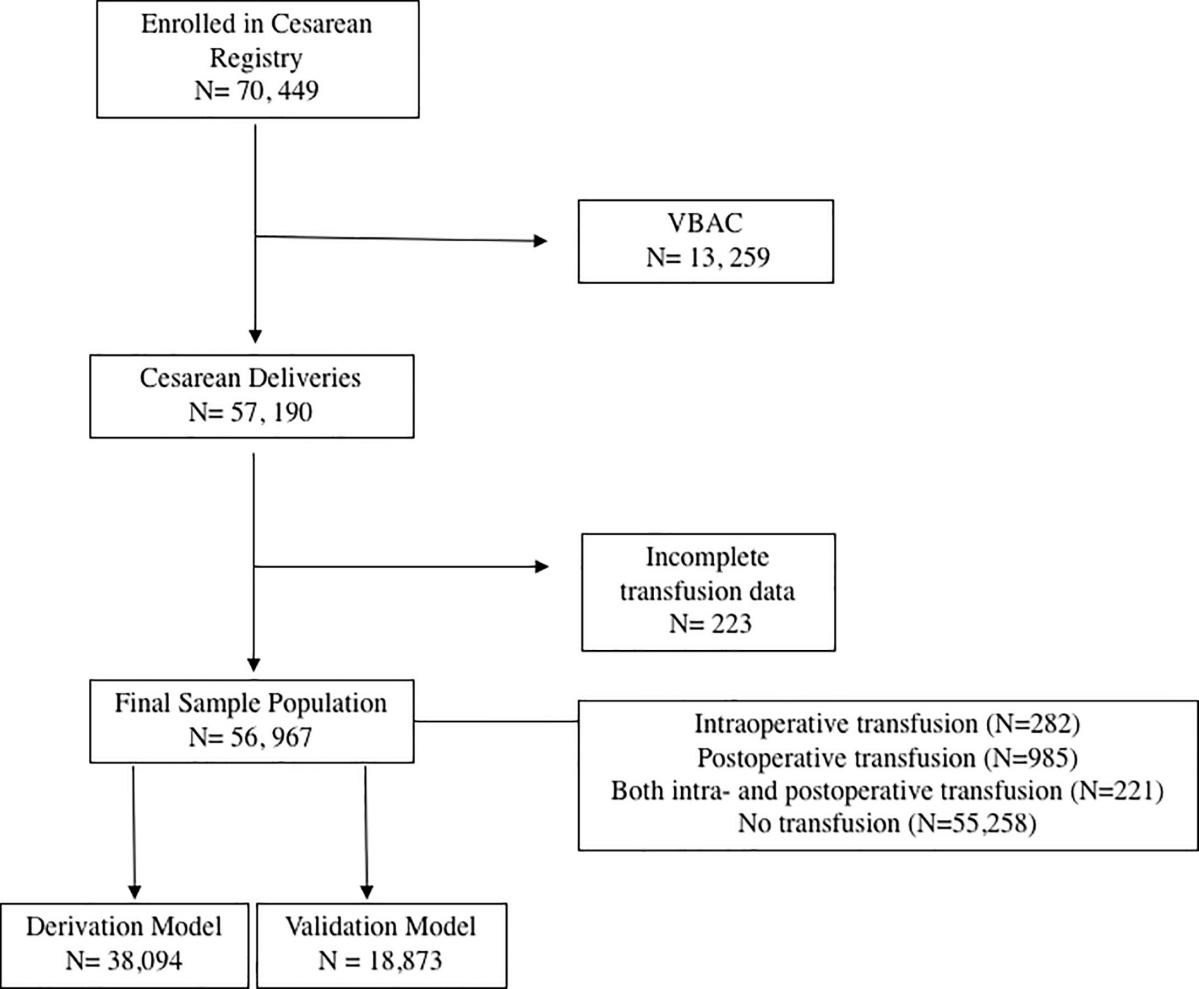
Flow diagram of women included in prediction model. Outlines who was included in the prediction model derivation and validation.

In the current analysis, patients were included who underwent CD. The primary goal for this analysis was to identify predictors of peripartum blood transfusion among women who underwent CD. Patients were excluded from the secondary analysis who had a vaginal delivery or incomplete data for both intrapartum and postpartum transfusion (**Fig 1**). Women considered as having the primary outcome in our analysis if they had intraoperative and/or postoperative blood transfusion.

### Candidate predictors

We included candidate predictors for the antepartum model based on prior known risk factors for postpartum hemorrhage and also availability in daily clinical practice[6,8,12,13]. Predictors for model 1 (antepartum risk factors) included the following: maternal age (extremes including age <21 or age >36), BMI (body mass index, defined as maternal height in meters divided by kilograms squared) at delivery, number of previous term deliveries, gestational age, gestational age >37 weeks, total years of schooling, African American ethnicity, insurance status for prenatal care, white blood cell count, platelet count, hematocrit, previous cesarean delivery, asthma, heart disease, connective tissue disorder, hypertensive disorder (gestational hypertension/preeclampsia/HELLP) and abruption. Predictors for model 2 (antepartum and intrapartum risk factors) included the same as model 1 plus the following: non-elective cesarean delivery, use of general anesthesia, abruption at the time of delivery, failure to progress (FTP), preeclampsia/eclampsia or HELLP, abnormal placentation and intrapartum antibiotic use (including treatment for chorioamnionitis or prophylaxis for GBS/cesarean delivery).

Lab values were obtained closest to admission. Suspected clinical abruption on presentation (antepartum) was distinguished separately from abruption as indication for cesarean delivery (intrapartum). FTP was collected as an indication for cesarean delivery and abnormal placentation (accreta/increta/percreta) clinically made diagnosis at time of delivery when removing placenta. Gestational hypertension and preeclampsia were considered antepartum risk factors while preeclampsia/eclampsia was considered an intrapartum risk factor. HELLP was classified both as an antepartum and intrapartum risk factor since this could be diagnosed in both time periods. To empirically determine whether the model improved upon the prediction accuracy that would be obtained by simply coding high risk based on known risk factors, we created a simple high-risk indicator based on the presence of preeclampsia, HELLP, low HCT, or abruption. Patients with any of these were considered “Hi-Risk”. Patients with none of these were considered “Low-risk”. (Only a few patients had more than one of these risk factors). We compared the area under the ROC curve (AUC) for this simple risk indicator with the AUC for our risk score based on our regression model, using a chi-square (Gonen M. Analyzing receiver operating characteristic curves with SAS 2007. Cary, NC, SAS Institute Inc.).

To test for possible sampling bias that could have been introduced by over-sampling women with repeat CD in the later years of data collection, we performed a sensitivity analysis in which we examined the performance of the pre-delivery model in the validation sample, using only women who gave birth during the years when repeat CD was not over-represented (1999–2000).

### Data analysis

We used 2/3 of cases, selected randomly, for the derivation sample (n = 38,094) and the remaining 1/3 (n = 18,873) for the validation sample. Two separate models were constructed, one model using only variables that would be known pre-delivery or antepartum, and the other model also including variables that would be known intrapartum. All model development steps were done using a complete-case analysis, only using the derivation sample. This included examining univariable associations of ante- and intra-partum variables with transfusion status, building multivariable models, testing for multicollinearity and determining the test performance at false positive rate of 10%. Multivariable logistic regression was used to examine prediction models, with backward elimination of predictors that had p>.20. Final selection of variables for the multivariable logistic regression was subsequently based on clinical relevance and ease with which it can be externally validated.

The final regression model was of the form: y = intercept + b_1_x_1_ + b_2_x_2_ + … b_n_x_n_, where the b_i_’s are the parameter estimates and the x_i_’s are the predictor values for each subject. The probability of peripartum transfusion was then defined as *p* = *e*^y^ / (1 + *e*^y^)[21]. Multicollinearity was tested using weighted regression, with any variance inflation factor > 2.0 indicating a problem (Allison, 1999). If this occurred, the collinearity diagnostic matrix was examined to identify overlapping variables so one of them could be eliminated. Since the intent is to use this marker in a general pre-delivery population, we wanted to maintain a low rate of false positives to minimize the number of women who would be subjected to unneeded interventions, while we could still identify a high proportion of those who might need such interventions. Therefore, we defined the test performance at false positive rate (FPR) of 10%, and we hoped to be able to identify at least 50% of those who would go on to have transfusions (true positive rate, TPR).

We then tested the resulting models in the validation sample, using a variety of tests for model replication. These included: a) the area under the receiver operating characteristic curve (AUC) using the probability estimate calculated using the above equation as a univariable predictor; b) the sensitivity and specificity using the test performance at false positive rate of 10%; and c) model calibration was tested in the validation sample by examining the association between predicted probability of transfusion and observed incidence in deciles of the risk score distribution. The risk of over-fitting was minimized by ensuring that k > 10n, where k = number of predictors in the model, and n = number of outcome events, and by using a separate validation sample. SAS (version 9.3, Cary, NC) was used for data analysis with a two-tailed p < .05 considered significant. No imputation for missing data was performed.

## Results

There were 56,967 cases with transfusion data available. 282 had intraoperative transfusion, 985 had postpartum transfusion, and 221 had both, for a total of 1488 (2.6%) with any transfusion. Due to the large sample size, differences were significant on almost all pre-surgery patient variables (**Table 1**). In the derivation sample (n = 38,094), 1002 mothers had peripartum transfusions (2.6%). Looking first at the antepartum predictors, those with transfusion were younger, more likely to be African American, less likely to be married, had lower BMI at delivery, had fewer years of school, were less likely to have had a previous CD, had more previous term deliveries, were more likely to have Medicaid or Medicare insurance, were more likely to have had previous VBACs, and were more likely to deliver babies with gestational age < 37 weeks. They had higher WBC and lower platelets and hematocrit, and were more likely to have preeclampsia, history heart disease, connective tissue disease, asthma, and these births were more likely to be multiples.

**Table 1:** Maternal variables by transfusion status in the derivation sample.

Maternal variables known ante-partum	Transfusion (n = 1002)	No Transfusion (n = 37,091)	p
Age at delivery (yrs)	27.9 ± 6.8	28.4 ± 6.2	0.02
Race/ethnicity			< .001
AA	410 (41)	10,144 (27)	
White	259 (26)	15,087 (41)	
Hispanic	277 (28)	9998 (27)	
Asian	23 (2)	655 (2)	
Other	33 (3)	1207 (3)	
Marital status			< .001
Married	451 (45)	21,298 (57)	
Not married	528 (53)	15,272 (41)	
Unknown	23 (2)	521 (1)	
BMI at delivery	31.9 ± 7.6	33.1 ± 7.2	< .001
Smoked during pregnancy	148 (15)	4959 (13)	0.19
Alcohol use during pregnancy	40 (4)	1221 (3)	0.22
Total years of schooling	11.7 ± 2.3	12.1 ± 2.5	< .001
Number of previous cesarean deliveries			< .001
0	506 (51)	14,991 (41)	
1	282 (28)	15,428 (42)	
2	136 (14)	5161 (14)	
3 or more	70 (7)	1373 (4)	
Number of previous term deliveries			< .001
0	142 (18)	5444 (18)	
1	252 (32)	13,767 (47)	
2	179 (23)	6721 (23)	
3 or more	214 (21)	3673 (12)	
Insurance for birth			< .001
Private	316 (32)	16,336 (44)	
Medicaid/Medicare	484 (48)	13,847 (37)	
None/self-pay	201 (20)	6902 (19)	
Insurance for prenatal care			< .001
Private	294 (32)	15,648 (44)	
Medicaid/Medicare	479 (52)	14,710 (42)	
None/self-pay	144 (16)	4932 (14)	
Number of previous VBAC			0.006
0	896 (93)	34,619 (95)	
1	38 (4)	1222 (3)	
2	16 (2)	325 (1)	
3 or more	9 (1)	163 (0.5)	
Any previous VBAC	63 (8)	1713 (6)	0.004
Gestational age at delivery (days)	254 ± 31	267 ± 22	< .001
Gestational age ≥ 37 weeks	568 (57)	29,742 (80)	< .001
WBC	10.7 ± 3.7	10.3 ± 2.9	0.001
Platelets	216.4 ± 70.6	222.9 ± 54.2	0.005
Hematocrit	31.6 ± 5.1	35.1 ± 3.6	< .0001
Asthma	93 (9)	2716 (7)	0.019
Preeclampsia	140 (14)	3217 (9)	< .001
Hx heart disease	24 (2)	458 (1)	0.001
Hx connective tissue disease	15 (1.5)	181 (0.5)	< .001
Multiple birth	70 (7)	1480 (4)	< .001
Sex of infant male	515 (51)	19,483 (53)	0.48
**Maternal and infant variables known intra-partum**			
Any oxytocin used	273 (27)	9472 (26)	0.22
Oxytocin time (hrs)	2.5 ± 5.3	2.2 ± 4.8	0.14*
Oxytocin maximum dose (mIU/min)	4.9 ± 10.5	3.9 ± 8.7	0.07*
General anesthesia	408 (41)	2571 (7)	< .001
Abruption	119 (12)	759 (2)	< .001
Placenta accreta, increta, or percreta	98 (10)	32 (0.1)	< .001
FTP	120 (12)	4005 (11)	0.24
Eclampsia or HELLP	44 (4)	323 (1)	< .001
Non-elective repeat CS	312 (31)	7931 (21)	< .001
Non-elective primary CS	514 (51)	15,124 (41)	< .0001
Intrapartum antibiotic administration	857 (86)	29,270 (79)	< .001

AA: African American, BMI: body mass index, VBAC: vaginal birth after cesarean, WBC: white blood cell count, FTP: failure to progress, HELLP: hemolysis, elevated liver enzymes, low platelets, CS: cesarean section

Data are mean ± standard deviation and n (%) unless otherwise specified

*Tested using non-parametric Kruskal-Wallis test due to skewed distribution; all other p-values based on the t-test for continuous variables or χ^2^ for categorical variables.

In the sensitivity analysis using only women who gave birth in 1999–2000, in the validation sample, there were 12,941 cases of which 12,918 with complete data were used in the analysis. 351 were positive for transfusion (2.7%). The probability computed from the regression model remained significantly associated with the outcome (p < .0001), with an OR of 2.52 (95% CI 2.28–2.77) and an AUC of 0.74 (95% CI 0.71–0.77). We divided the distribution of risk scores into deciles, and examined the incidence of transfusion across deciles. The incidence was 0.8% in the lowest decile (10 cases out of 1291) and 11% in the highest decile (136 cases out of 1292). This suggests that there was little sampling bias due to differences across years.

### Model 1 antepartum model

**Fig 2:**
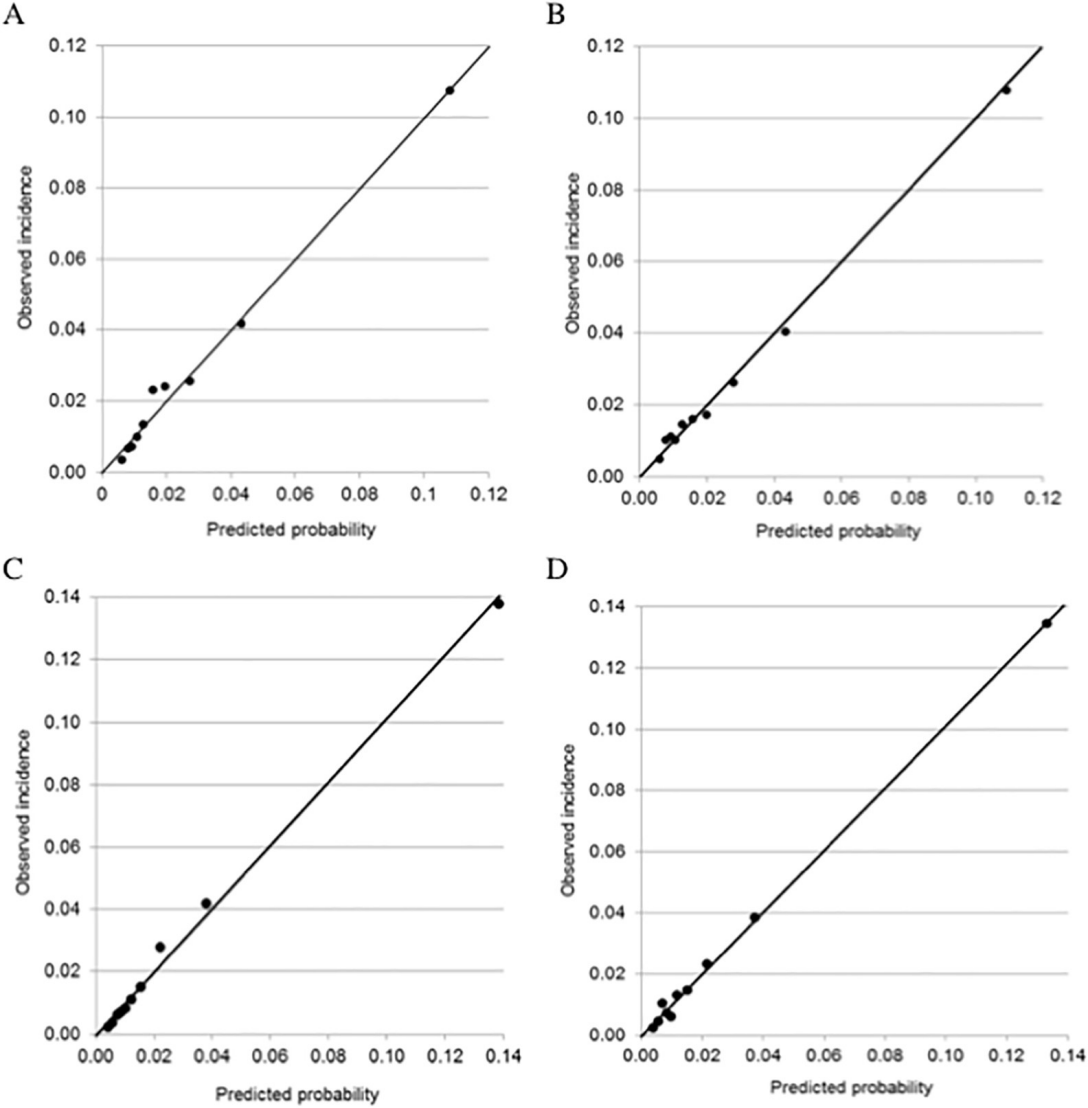
Calibration curves for Model 1 and Model 2. Model 1 includes antepartum risk factors only and Model 2 includes antepartum and intrapartum risk factors. **a. Model 1—Derivation Sample.** Calibration of the model predicting transfusion using only antepartum variables. **b. Model 1—Validation Sample.** Calibration of the model predicting transfusion using only antepartum variables.

The final multivariable model predicting perinatal transfusion in the derivation sample had AUC = 0.77 (95% confidence interval 0.75–0.78), indicating moderate discrimination. Variance inflation factors were all <1.8. Predictors that were retained in the final model included mother’s age (coded as either <21 or >36, versus 21–36), race, BMI, type of insurance, presence of abruption, platelet count < 150 x 10^3^ units, hematocrit < 32%, presence of gestational HTN or preeclampsia, presence of HELLP, history of asthma, history of heart disease, gestational age < 37 weeks, and 3 or more previous term deliveries (versus <3) (**Table 2**). The calibration graph indicated good agreement of predicted probability and observed incidence (**Fig 2A**). However, the highest risk decile only had incidence of 0.11 and the risk deciles were not evenly distributed. An FPR of 0.10 was achieved using a probability cut point of p ≥ 0.055, and at this cut point the TPR was 0.42. At the other end of the probability distribution, using a cut point of 0.01, TPR of 0.92 was achieved with the corresponding FPR of 0.68. The predicted chance for peripartum transfusion can be calculated for women undergoing cesarean delivery using the following formula:

$$\begin{array}{l} y=‑4.6841\;+.2301*\left(age<21\;or\;>36\right)\\ \;‑.0138*\left(body\;mass\;index\;or\;BMI\right)\\ \;+.5708*\left(platelets<150\right)\\ \;+1.3125*\left(hematocrit<32\right)\\ \;+.2065*\left(gest.HTN\;or\;preeclampsia\right)\\ \;+1.1069*\left(HELLP\right)\\ \;+.1496*\left(hx\;asthma\right)\\ \;+1.1821*\left(abruption\right)\\ \;+.2369*\left(African\;American\right)\\ \;+.3122*\left(Medicare\;or\;Medicaid\right)\\ \;+.4107*\left(No\;insurance\right)\\ \;+.6127\left(gestational\;age\;<37\;wks\right)\\ \;+.6538*\left(3\;or\;more\;previous\;term\;deliveries\right)\\ \;+.7678*\left(heart\;disease\right)\\ \;+.4439(first\;CD)\\ \;+.3029\left(2\;previous\;CD\right)\\ \;+.4526\left(3\;or\;more\;previous\;CD\right) \end{array}$$

**Table 2:** Multivariable models for predicting perinatal transfusion.

	Model 1^a^ (n = 38,005)					Model 2^b^ (n = 37,930)				
Characteristic	Parameter Estimate	Odds Ratio	OR 95% Confidence Limits		p-value	Parameter Estimate	Odds Ratio	OR 95% Confidence Limits		p-value
Intercept	-4.68				< .0001	-5.24				< .0001
**Antepartum**										
Maternal age <21 or >36, years	0.23	1.26	1.09	1.46	.002	0.16	1.17	1.00	1.37	.04
BMI at delivery, kg/m^2^	-0.014	0.99	0.98	0.99	.007	-0.0153	0.99	0.98	0.99	.004
3+ previous term deliveries	0.65	1.92	1.60	2.34	< .0001	0.6342	1.89	1.55	2.29	< .0001
Gestational age <37 weeks	0.63	1.85	1.59	2.14	< .0001	-	-	-	-	-
African American race	0.24	1.27	1.10	1.47	.002	-	-	-	-	-
Insurance: govt vs. private	0.31	1.37	1.17	1.60	.0001	0.27	1.32	1.12	1.55	.0009
Insurance: none vs. private	0.41	1.51	1.25	1.82	< .0001	0.23	1.25	1.02	1.53	.03
Platelet count <150, (x10^3^)	0.57	1.77	1.45	2.17	< .0001	0.37	1.45	1.17	1.80	.0008
Hematocrit <32, (%)	1.31	3.72	3.25	4.25	< .0001	1.31	3.71	3.22	4.27	< .0001
No previous CD	0.44	1.56	1.34	1.82	< .0001	0.61	1.83	1.45	2.32	< .0001
2 previous CD	0.31	1.35	1.10	1.67	.005	0.41	1.50	1.19	1.90	.0007
3+ previous CD	0.45	1.57	1.16	2.13	.003	0.18	1.21	0.84	1.74	.32
Hx asthma	0.15	1.16	0.93	1.46	.20	-	-	-	-	-
Hx heart disease	0.77	2.16	1.40	3.32	.0005	0.75	2.12	1.35	3.34	.001
Gest. HTN/Preeclampsia	0.21	1.23	1.01	1.50	.04	0.35	1.42	1.18	1.71	.0003
HELLP	1.11	3.03	2.00	4.57	< .0001	-	-	-	-	-
Abruption antepartum	1.18	3.26	2.60	4.10	< .0001	-	-	-	-	-
**Intrapartum**										
Non-elective repeat CD	-	-	-	-	-	0.54	1.72	1.39	2.14	< .0001
General anesthesia	-	-	-	-	-	1.65	5.16	4.44	6.08	< .0001
Abruption at time of delivery	-	-	-	-	-	0.96	2.61	2.06	3.32	< .0001
Multiple gestational	-	-	-	-	-	0.55	1.73	1.32	2.26	< .0001
Failure to progress	-	-	-	-	-	0.50	1.65	1.32	2.08	< .0001
Eclampsia/HELLP						1.06	2.87	1.96	4.21	< .0001
Placenta accreta, increta, or percreta	-	-	-	-	-	4.52	92.02	57.36	147.63	< .0001
Antibiotic use	-	-	-	-	-	0.28	1.33	1.09	1.61	.004

^a^Model 1: antepartum risk factors; reference group for previous CD = 1

^b^Model 2: antepartum and intrapartum risk factors

When compared with assignment of high-risk status based on known risk factors, we found that while the risk score calculated from our model had AUC 0.75 (95% CI 0.33–0.78), the simpler risk model had AUC of 0.59 (0.57–0.61), a difference that was significant (p < .0001). Therefore, the model we propose significantly improves prediction accuracy compared with using a simpler approach to risk stratification based on a few known risk factors.

In the validation sample the incidence of transfusion was also 2.6%. When the risk equation was used in the validation sample as the only predictor of transfusion, the AUC for the univariable logistic regression model was 0.75 (95% CI 0.73–0.78). Using *p* = 0.055 as the cut point for predicting transfusion in the validation sample, the TPR was 0.43 with FPR of 0.10. The model remained well-calibrated (**Fig 2B**). In the validation sample, the observed incidence of transfusion was 10.8% in mothers with the highest probability decile and 0.5% in mothers with the lowest probability decile. Mean predicted probability of transfusion in deciles 10 and 1 was 10.9% (95% CI 10.6–11.2) and 0.6% (95% CI 0.60–0.61), respectively.

### Model 2 antepartum and intrapartum model

Variables that would only be known during the delivery process, that were added to the Intra-partum model included general anesthesia, placenta accreta, eclampsia, use of oxytocin, intrapartum decision for CD, and intra-partum use of antibiotic. All of these were more common in the group with transfusions (Table 1). The final multivariable intrapartum prediction model had AUC = 0.83 (95% CI 0.81–0.84) in the derivation sample, and included the intrapartum predictors general anesthesia, FTP, eclampsia, accreta/increta/percreta, antibiotic use, and non-elective repeat CD (**Table 2**). All variance inflation factor scores were < 1.75. The predicted chance for peripartum transfusion can be calculated for women undergoing cesarean delivery using the following formula:

$$\begin{array}{l} y=‑5.2402\;+0.1583*\left(age<21\;or\;>36\right)\\ \;‑0.0153*\left(body\;mass\;index\;or\;BMI\right)\\ \;+0.3706*\left(platelets<150\right)\\ \;+1.3111*\left(hematocrit\;<32\right)\\ \;+0.3479*\left(gestational\;HTN\;or\;preeclampsia\right)\\ \;+1.0587*\left(eclampsia\;or\;HELLP\right)\\ \;+0.8218*\left(abruption\right)\\ \;+0.3313*\left(abruption\;was\;an\;indication\;for\;CD\right)\\ \;+0.5894*\left(no\;previous\;CD\right)\\ \;+0.4069*\left(2\;previous\;CD\right)\\ \;+0.2773*\left(Medicare\;or\;Medicaid\right)\\ \;+0.2258*\left(no\;insurance\right)\\ \;+0.6304*\left(3\;or\;more\;previous\;term\;deliveries\right)\\ \;+0.7524*\left(hx\;of\;heart\;disease\right)\\ \;+1.6439*\left(general\;anesthesia\right)\\ \;+0.5205*\left(failure\;to\;progress\;or\;FTP\right)\\ \;+4.5154*\left(accreta/increta/percreta\right)\\ \;+0.2802*\left(used\;intra‑partum\;antibiotics\right)\\ \;+0.5607*\left(non‑elective\;repeat\;CD\right)\\ \;+0.5518\left(multiple\;birth\right) \end{array}$$

The model calibration graph in the derivation sample indicated good agreement between predicted probability and observed incidence of peri-partum transfusion (**Fig 2C**). However, again, the risk deciles clustered toward the lower end of the probability distribution rather than being spread out evenly. The incidence of transfusion in the highest risk decile was 13.8%, versus 0.2% in the lowest decile. Using a cut point of *p* = 0.047, TPR was 0.55, while FPR was 0.10. In the validation sample, AUC for this model was 0.82 (95% CI 0.79–0.84), indicating good discrimination. Model calibration continued to be good (**Fig 2D**). Using the cut point of *p* = 0.047, the FPR in the validation sample was 0.10 with TPR 0.54. In other words, for the intra-partum model, the probability cut point generated from the derivation data produced nearly identical results in the validation sample.

Among the patients with asthma who had uterine atony but no transfusion (n = 1,074), 8.5% received methergine versus 4.0% received hemabate (p = 0.004). More specifically, among the patients with asthma who had uterine atony with transfusion (n = 164), 19.4% received methergine versus 8.8% received hemabate (p = 0.05).

## Discussion

### Main findings

In this study, we have built a prediction model that accurately estimates a patient’s odds of receiving blood transfusion during or after a CD. Our results demonstrate a distinct group of risk factors that predispose women for peripartum blood transfusion, a clear objective marker signifying morbidity. In our model, internal validation had good discrimination and validation. Our observed rate of transfusion is 2.6% (1,488 / 56,967 * 100), slightly above the previously reported overall rate when combining vaginal and cesarean deliveries[13,22,23]. Our rate is comparable to previously observed rates among CD, which have been reported between 3.2–3.3%[23–25].

In a study by Helman *et al*, risks factors associated with severe PPH (defined as 5 or more units of pRBCs) were comparable with our findings, including abnormal placentation, hypertensive disorders, and multifetal gestation[16]. Though extensive research has been done evaluating risk factors for severe hemorrhage, our model is novel in that we are attempting to isolate individuals who are at highest likelihood of transfusion prior to delivery. In a previous study by Bateman *et al*, looking at the risk for any transfusion during vaginal or cesarean delivery, they were unable to identify risk factors with good predictability or discrimination (ROC < 0.7). However, they did identify similar risk factors to those in our study such as advanced maternal age, hypertensive disease and multiple gestations[12]. The explanation for the differences in the model accuracies is likely attributable to the level of detail provided in the MFMU Cesarean Registry compared with the Nationwide Inpatient Sample.

We observed that deliveries at an earlier gestational age, and correspondingly with lower birthweight, had a significantly increased odds for massive transfusion. Although we did not use birthweight as a variable in the risk prediction model, it is highly correlated with gestational age. Mothers who have more comorbidities such as hypertension or diabetes are more likely to have preterm deliveries, lower birthweight, and other risk factors that contribute to obstetric hemorrhage[9,12,16,18].

One of the most significant risk factors for blood transfusion is preoperative anemia[9,14,17]. We found preoperative anemia (hematocrit <32%) also to be a significant predictor for need of transfusion (OR 3.72, 95% CI 3.25–4.25). In a study by Al-Zirqi, *et al*, severe anemia with a hemoglobin < 9.0 g/dL was associated with an increased odds of transfusion (OR 2.2; CI 1.63–3.15)[17]. Another study by Ehrenthal, *et al*, demonstrated that a starting hemoglobin < 10.5 g/dL was associated with some risk of transfusion where a hemoglobin < 9.5 g/dL was associated with considerable risk[9].

Given that blood transfusion at delivery is a relatively rare occurrence, most women identified at risk by the model will likely not have the outcome. The true positive rate of at least 50% was achieved by model 2 with a small margin although not achieved by model 1, which suggests that the additional variables included in model 2 improved the detection ability. In terms of the utility of the model given the relatively high false positive rate, that all depends on how providers use this information. If used for delivery preparedness or consideration for prophylactic medications to reduce bleeding, whether for clinical or research purposes, this model still can be useful. Further improvement of the model will likely require larger sample sizes.

The current analysis differs from published literature in that it recognizes one prior CD is associated with lower odds for hemorrhage. Among patients with CD, a patient with a prior CD is more likely to have an elective or scheduled CD rather than an emergency or non-elective repeat CD. Many prior studies find that a prior CD increases a patient’s risk for severe obstetric hemorrhage[6,9,12,17]. Al-Zirqi, *et al* found that emergency CD was the most significant risk factor for severe obstetric hemorrhage (OR of 4.75; CI 1.32–12.96)[17]. We suspect our finding is due to the fact emergency CD is preceded by labor, either spontaneous or induced (which is becoming increasingly more common with the known complication of prolonged induction), possibly FTP, possibly prolonged oxytocin use and possibly chorioamnionitis, which increase the risk for uterine atony and postpartum hemorrhage[15,16].

Furthermore, the developed algorithm offers an objective way to consider use of prophylactic interventions that are being studied in research protocols such as antifibrinolytic therapy (i.e. tranexamic acid use) prior to delivery or immediately after delivery of the baby. Utilization a validated algorithm for prophylactic use of a medication would be more acceptable than consideration of only clinical risk factors left up to clinical discretion.

### Strengths and limitations

A strength of this study is the ability to analyze a large cohort of primary and repeat CD, making it generalizable to a large number of patient populations. Predictors included within the model are factors that are often identifiable in perioperative setting, making the model useful and applicable in every day practice. In addition, our model allows for identification of patients who are at increased risk of transfusion in the perioperative setting, which could allow for pre-delivery optimization. For example, this could include aggressive management of pre-delivery anemia with intravenous iron and/or transfusion. Furthermore, in rural regions that are not as equipped with transfusion medicine services or subspecialty surgical services, consideration for transfer to higher level of care should be given when the prediction model estimates a higher than baseline risk for transfusion (assuming stable condition for patient prior to delivery).

Some limitations of our study include the retrospective aspect of the data and a focus that exclusively evaluates hemorrhage and transfusion in the setting of CD, which does not provide direct insight to similar complications that arise during a vaginal delivery. Another important limitation of the model is a possibility to underestimate the risk of transfusion in the setting of an unexpected outcome or evolving clinical situation. For example, encountering an undiagnosed accreta or abruption, or an unexpected conversion to general anesthesia. Furthermore, for some predictors it is not possible to evaluate temporal associations (i.e. was general anesthesia a predictor of hemorrhage requiring transfusion or the result after transfusion initiated). Although our model demonstrated good discrimination during internal validation, important next steps include external validation of the model by using another cohort of patients. Finally, an optimal threshold for risk of transfusion should be based on further risk-benefit analysis and was beyond the scope of our study.

### Interpretation

This model has promising clinical use in identification of patients who are at increased risk of transfusion during or after CD. This offers a unique opportunity for providers to improve delivery preparedness for both patients and patient care teams. In the office, patient counseling could increase awareness of potential complications at time of delivery. In addition, setting patient expectations and allowing opportunities for providers to answer questions could potentially increase patient preparedness and decrease the emotional trauma that is commonly associated with sentinel maternal events like massive hemorrhage[26]. Our risk calculator using the key antepartum and intrapartum factors can be found on https://www.gwdocs.com/mfm/peripartum-prediction-of-blood-transfusion.

## Conclusion

Using this model to risk stratify CD, patient care teams can implement strategies for risk reduction including cross matching for units of blood and having second level uterotonic medications or extra personnel available in the operating room. Also, this model could be considered to stratify who should be considered for prophylactic use of alternative hemostatic agents such as tranexamic acid. External validation of the model using a different cohort of patients will add to the generalizability of the model.

In summary, with an accurate prediction model, those undergoing cesarean delivery who are at risk for severe PPH requiring transfusion could be identified prior to delivery which may improve obstetric team preparedness, preoperative planning, and patient counseling.
