# Correction: Predicting peripartum blood transfusion in women undergoing cesarean delivery: A risk prediction model

**DOI:** 10.1371/journal.pone.0211360

**Published:** 2019-01-23

**Authors:** Homa K. Ahmadzia, Jaclyn M. Phillips, Andra H. James, Madeline M. Rice, Richard L. Amdur

There is an error in the link included in the last sentence of the “Interpretation” sub-section of the Discussion. The correct link is: http://www.gw-docs.com/mfm/peripartum-prediction-of-blood-transfusion.
